# Supplementary figures and images for: A monoclonal antibody raised against Acinetobacter baumannii capsular carbohydrate exhibits cross-species in vitro binding against Pseudomonas aeruginosa
Source: PLoS One. 2026 Jan 12;21(1):e0340857. doi: 10.1371/journal.pone.0340857 (PMC12795346; doi:10.1371/journal.pone.0340857)

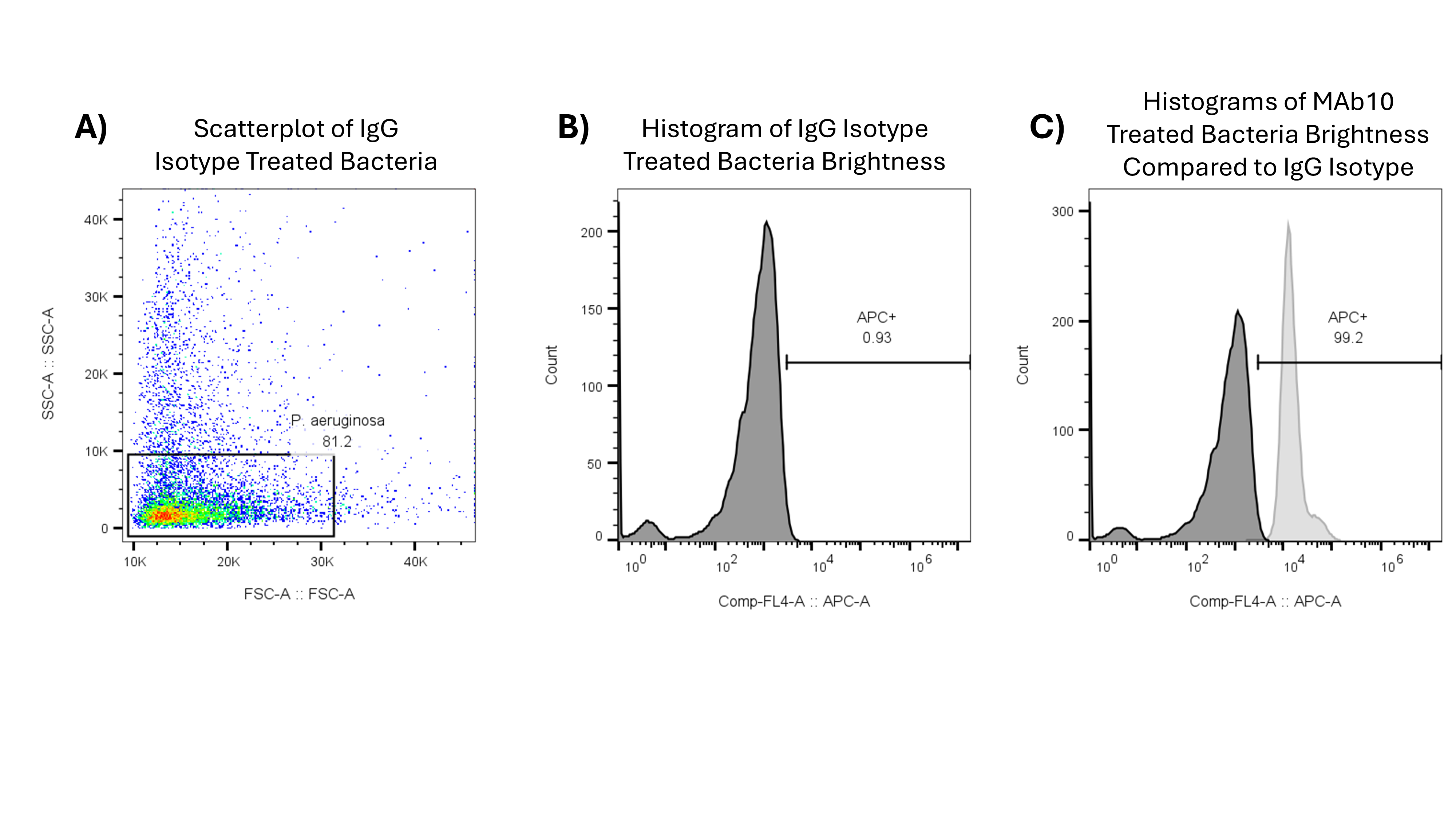

Supplement: S1 Fig — Flow cytometry events from the IgG isotype control treated group were first viewed on a forward and side scatter plot. Excessively large or granular events were eliminated via box gate (A). Events selected via gating in figure A were then plotted as a histogram with the X axis displaying brightness on the flow cytometer’s APC channel. A gate was used to select any events brighter than the 99th percentile of the IgG isotype control (B). This gate was then applied to the MAb treated group, and the percentage of events that fell within that gate were interpreted as the ‘percent binding’. In the sample shown, the percent binding is 99.2% (C). (TIF) [file pone.0340857.s001.tif]
