# Supplementary material for: A monoclonal antibody raised against Acinetobacter baumannii capsular carbohydrate exhibits cross-species in vitro binding against Pseudomonas aeruginosa
Source: PLoS One. 2026 Jan 12;21(1):e0340857. doi: 10.1371/journal.pone.0340857 (PMC12795346; doi:10.1371/journal.pone.0340857)
Supplement: Attachment — Submitted filename: PlosONE response to reviewers.docx [file pone.0340857.s004.docx]

**Reviewer #1:**

1. **I recommend publication after minor editorial revisions.**

We thank you for your positive assessment of our work.

**Reviewer #2:**

1. **The novelty is limited since the lack of in vivo efficacy diminishes translational impact. Authors should better justify the originality of this work relative to prior studies**
   a. The rational approach to the characterization of the MAb, including the shared capsule features across species, has not been previously reported. It was therefore highly encouraging that our hypothesis was correct and that a MAb that was raised against *A. baumannii* was also able to bind to *P. aeruginosa.* Though we had hoped to see *in vivo* efficacy of this antibody against *P. aeruginosa* that was comparable to the extensive efficacy that was previously demonstrated against *A. baumannii*, we feel that the lack of efficacy against *P. aeruginosa* makes for an important scientific observation. The differential in efficacy of the MAb in these distinct species despite similar *in vitro* observations between the two demonstrates the challenge of producing an antimicrobial MAb that is able to treat infections caused by multiple pathogens. This in itself is the point we aim to make with this paper.
2. **The rationale for testing P. aeruginosa appears speculative. A more systematic antigenic or epitope-level comparison should precede experimental testing to justify this direction.**
   a. The initial hypothesis was informed by the finding that MAb10 was able to bind only to *A. baumannii* strains that expressed capsule types that contained pseudaminic acid residues, with great consistency across a panel of 550 clinical isolates – data that was published in the paper that initially characterized MAb10. With this in mind, testing the MAb via flow cytometry against *P. aeruginosa* strains that were known to express pseudaminic acid was a simple and direct experiment, the results of which naturally led to the subsequent efficacy screens. Consistent with our hypothesis, the MAb was able to bind well to *P. aeruginosa* in vitro.
3. **The central hypothesis—that MAb10 binds pseudaminic acid-containing epitopes across species—requires biochemical validation. Without structural confirmation (e.g., glycan array or ELISA with purified carbohydrates), this remains speculative.**
   a. Given the lack of efficacy of MAb10 against *P. aeruginosa*, we do not feel that further investigation into the precise epitope that MAb10 binds on this species is warranted. Our intent is to publish the finding that MAb10 binds to both species (potentially because of their shared expression of pseudaminic acid) but only shows efficacy against one. The discussion section describes this limitation.
4. **Also, authors must discuss alternative explanations for cross-reactivity, such as nonspecific binding or shared glyco-motifs unrelated to pseudaminic acid.**
   a. This has been added to the discussion as requested (line 92)
5. **The in vitro binding results rely solely on flow cytometry. There is no orthogonal confirmation (e.g., ELISA, immunoblot, or surface plasmon resonance).**
   a. Given the lack of translational impact of MAb10 against *P. aeruginosa*, we do not feel that such experiments are warranted at this time.
6. **The use of only one or two P. aeruginosa reference strains (O7 and O9) limits generalizability. Broader strain diversity should be analyzed or statistically summarized.**
   a. We have added an additional reference strain which lacks pseudaminic acid, and which MAb10 demonstrably does not bind to. Furthermore, there is a broad screen of 118 clinical isolates of *P. aeruginosa* included as a heatmap in figure 1E, wherein MAb10 bound to 25 of them.
7. **The opsonophagocytosis assay shows marginal significance (p = 0.0476) and a modest biological effect. Replication and quantitative statistical reporting (effect size, SD, n) are needed.**
   a. The figure has been updated to clarify that it is representative of two experiments with similar outcomes. The quantitative and statistical reporting has been added to the figure legend as requested.
8. **The manuscript lacks proper negative and positive controls for cross-reactivity—e.g., known P. aeruginosa O-antigen MAb as a comparator.**
   a. To our knowledge, no such positive control is presently available. A strain with a verified serotype that lacks pseudaminic acid has been added as a negative control, and is now in figure 1D.
9. **Antibody purity, concentration verification, and endotoxin levels should be described since these can affect macrophage responses and animal outcomes.
   There is no mention of antibody affinity or subclass, both of which influence effector function and could explain the lack of protection.**
   a. This information has been added to the methods section.
